# Bifurcation analysis and new waveforms to the first fractional WBBM equation

**DOI:** 10.1038/s41598-024-62754-0

**Published:** 2024-05-24

**Authors:** Mohammad Safi Ullah, M. Zulfikar Ali, Harun-Or Roshid

**Affiliations:** 1 Department of Mathematics, Comilla University, Cumilla, 3506 Bangladesh; 2 Department of Mathematics, University of Rajshahi, Rajshahi, 6205 Bangladesh; 3 Department of Mathematics, Pabna University of Science and Technology, Pabna, 6600 Bangladesh

**Keywords:** Stability analysis, Chaotic nature, Soliton, Sensitivity analysis, Solitons, Nonlinear phenomena

## Abstract

This research focuses on bifurcation analysis and new waveforms for the first fractional 3D Wazwaz–Benjamin–Bona–Mahony (WBBM) structure, which arises in shallow water waves. The linear stability technique is also employed to assess the stability of the mentioned model. The suggested equation’s dynamical system is obtained by applying the Galilean transformation to achieve our goal. Subsequently, bifurcation, chaos, and sensitivity analysis of the mentioned model are conducted by applying the principles of the planar dynamical system. We obtain periodic, quasi-periodic, and chaotic behaviors of the mentioned model. Furthermore, we introduce and delve into diverse solitary wave solutions, encompassing bright soliton, dark soliton, kink wave, periodic waves, and anti-kink waves. These solutions are visually presented through simulations, highlighting their distinct characteristics and existence. The results highlight the effectiveness, brevity, and efficiency of the employed integration methods. They also suggest their applicability to delving into more intricate nonlinear models emerging in modern science and engineering scenarios. The novelty of this research lies in its detailed analysis of the governing model, which provides insights into its complex dynamics and varied wave structures. This study also advances the understanding of nonlinear wave properties in shallow water by combining bifurcation analysis, chaotic behavior, waveform characteristics, and stability assessments.

## Introduction

Nonlinear evolution equations play an important role in nonlinear science owing to their wide variety of applications. These equations describe many complex phenomena, including fluids^1,2^, human diseases^3^, heat transfer mechanism^4–6^, shallow water wave^7^, magnetohydrodynamics^8,9^, aerodynamics^10^, plasma physics^11–13^, molecular biology^14^, telecommunications^15^, nonlinear optics^16,17^, condensed matter physics^18,19^, and more^20–22^. Therefore, soliton solutions to nonlinear models are highly attractive^23,24^. The existence of these soliton outcomes is demonstrated in many distinguished nonlinear models like the Jimbo–Miwa equation^25^, Phi-4 model^26^, the Konopelchenko–Dubrovsky system^27^, the fractional 3D WBBM model^28^, the Fokas-Lenells model^29^, the LPD equation^30^, Kundu–Mukherjee–Naskar model^31^, predator–prey equation^32^, and various others^33–35^. Several effective methodologies exist for solving these nonlinear models and obtaining soliton results. These approaches encompass the expansion technique involving the Kudryashov approach^36^, the unified scheme^37^, the modified extended tanh approach^38^, the $(G'/G,1/G)$ algorithm^39^, the Hirota bilinear process^40^, the $(\frac{G'}{G^2})$ expansion process^41,42^, the extended ShGEEM methodology^43^, and the references therein^44–46^.

Bifurcation analysis is a powerful tool in the study of dynamic systems and holds significant implications across diverse domains^47–49^. Liu and Li^50^ introduced the bifurcation method in 2002, a potent tool for investigating the dynamic properties of partial differential equations. It is especially effective in analyzing bifurcation phenomena and obtaining exact traveling wave solutions. It examines how a system’s qualitative behavior changes by varying its parameters^51^. This analysis helps researchers understand how systems transition between stable and unstable states or chaotic behavior. The key objective of this writing is to investigate bifurcation analysis and new waveforms within the first fractional 3D-WBBM^28^ nonlinear framework.

This research addresses a gap in understanding the behavior of the first fractional WBBM equation in shallow water wave dynamics, which has applications in coastal engineering, tsunami forecasting, and oceanic modeling. This study offers insights into the complex dynamics of these waves through advanced analytical and computational methods, including dark solitons, bright solitons, periodic waves, and kink waves. These findings enhance predictive models and decision-making processes in related fields, which significantly contribute to advancing knowledge of nonlinear wave dynamics and its practical applications.

By applying the techniques proposed for Eq. (7), this study provided novel findings that had not formerly been reported.

The structure of the existent investigation is outlined as follows: “Conformable derivative and its features” section contains the conformable derivative and its features. In “Suggested model” and “Ordinary differential form of the suggested model” sections cover the descriptions and the ordinary differential form of the governing model, respectively. Bifurcation analysis is detailed in “Bifurcation analysis” section. In “Chaotic behaviors” section, the chaotic natures of the stated model are presented. In “Sensitivity analysis” section encompasses the sensitivity analysis of the suggested model. In “Bright and dark solitons of the WBBM model” section provides dark and bright solitons of the mentioned model. The discussion of the results is contained in “Discussion of results” section. Stability analysis is given in “Stability analysis” section. In “Novelty of the outcomes” section discusses the novelty of the outcomes. Lastly, “Conclusion” section encapsulates the findings drawn from this study.

## Conformable derivative and its features

If we assume a real function $q:[0,\infty ]\rightarrow {\mathbb {R}}$, then for all $t>0$ and $\alpha \in (0,1]$, the conformable fractional derivative of *q*(*t*) takes the next form^55^:

1 $$\begin{aligned} & D_t^{\alpha }q(t)=\lim _{\epsilon \rightarrow 0}\frac{q(t+\epsilon t^{1-\alpha })-q(t)}{\epsilon }. \end{aligned}$$

Now, we describe some remarkable features of fractional derivatives. Let *q*(*t*) and *r*(*t*) are $\alpha$-conformable differentiable whilst $t>0$ with $\alpha \in (0,1]$. Then

(i) $$D_t^{\alpha }t^n=nt^{n-\alpha }$$ for all $$n \in {\mathbb {R}}$$
(ii) $D_t^{\alpha }(c)=0$ for any constant *c*.
(iii) $D_t^{\alpha }aq(t)=aD_t^{\alpha }q(t)$ with real constant *a*.
(iv) $D_t^{\alpha }(aq(t)+br(t))=aD_t^{\alpha }q(t)$+$bD_t^{\alpha }r(t)$ with real constants *a*, *b*.
(v) $$D_t^{\alpha }(r(t)q(t))=r(t)D_t^{\alpha }q(t)$$+$$q(t)D_t^{\alpha }r(t)$$.
(vi) $$D_t^{\alpha }(\frac{q(t)}{r(t)})=\frac{r(t)D_t^{\alpha }q(t)-q(t)D_t^{\alpha }r(t)}{r^2(t)}, r(t) \ne 0$$.
(vii) $D_t^{\alpha }(q(t))=t^{1-\alpha }\frac{dq}{dt}$ when *q*(*t*) is differentiable.

## Suggested model

The BBM equation was first observed in 1972 as a development of the KdV equation for shallow waves of water in a homogeneous system. The BBM equation is frequently used as a variant of the KdV equation for describing shallow water waves. It is applicable not only to surface waves in water but also to drift waves and Rossby waves in plasma within spinning environments. The BBM nonlinear model^52^ is given in the following manner:

2 $$\begin{aligned} & q_t+q_x+q^nq_x-q_{xxt}=0, \end{aligned}$$

and the KdV equation is given by

3 $$\begin{aligned} & q_t+q_x+qq_x+q_{xxx}=0, \end{aligned}$$

Both the BBM and KdV equations serve as foundational tools for comprehending various wave phenomena. They serve as essential tools for analyzing surface waves in water bodies, hydromagnetic waves in plasma, acoustical and gravitational waves in compressible fluids, acoustical waves in harmonic crystals, and long waves in nonlinear dispersive processes, among other applications. A new equation called the Wazwaz–Benjamin–Bona–Mahony (WBBM) equation^53^ was derived by Wazwaz in 2017 from a 3-dimensional modified BMM equation, which is expressed as

4 $$\begin{aligned} & q_t+q_x+q^2q_y+q_{xzt}=0, \end{aligned}$$

5 $$\begin{aligned} & q_t+q_z+q^2q_x+q_{xyt}=0, \end{aligned}$$

6 $$\begin{aligned} & q_t+q_y+q^2q_z+q_{xxt}=0. \end{aligned}$$

Wazwaz reformulated these newly introduced equations. This article will focus on the first fractional 3D WBBM equation, as presented in^54^, which is given in the following manner:

7 $$\begin{aligned} & D_t^{\alpha }q+D_x^{\alpha }q+D_y^{\alpha }q^3-D_{xzt}^{3\alpha }q=0, \end{aligned}$$

where *q*(*t*, *x*, *y*, *z*) is a wave function with 4 free components *t*, *x*, *y*, and *z*. $D_t^{\alpha }, D_x^{\alpha }, D_y^{\alpha }$, and $D_z^{\alpha }$ correspond to the fractional derivatives of order $\alpha$ w.r. to *t*, *x*, *y*, and *z*, sequentially whilst $0<\alpha \le 1, t \ge 0$.

## Ordinary differential form of the suggested model

Consider the next traveling wave relation for Eq. (7)

8 $$\begin{aligned} & q(x,y,z,t)=Q(\theta ),\theta = \frac{a_1}{\alpha }x^\alpha +\frac{a_2}{\alpha }y^\alpha +\frac{a_3}{\alpha }z^\alpha -\frac{a_4}{\alpha }t^\alpha , \end{aligned}$$

with real constants $a_1, a_2, a_3$, and $a_4$. Applying Eq. (8) in Eq. (7) gives the next outcome as

9 $$\begin{aligned} & (-a_4+a_1)Q'+a_2(Q^3)'+a_1a_3a_4Q'''=0. \end{aligned}$$

Integrating Eq. (9) w. r. to $\theta$, one obtains

10 $$\begin{aligned} & (-a_4+a_1)Q+a_2Q^3+a_1a_3a_4Q''+a_5=0, \end{aligned}$$

with integration constant $a_5$. For simplicity $a_5$, by setting $a_5$ to 0, one reaches the next ordinary differential form as

11 $$\begin{aligned} & (-a_4+a_1)Q+a_2Q^3+a_1a_3a_4Q''=0, \end{aligned}$$

**Figure 1:**
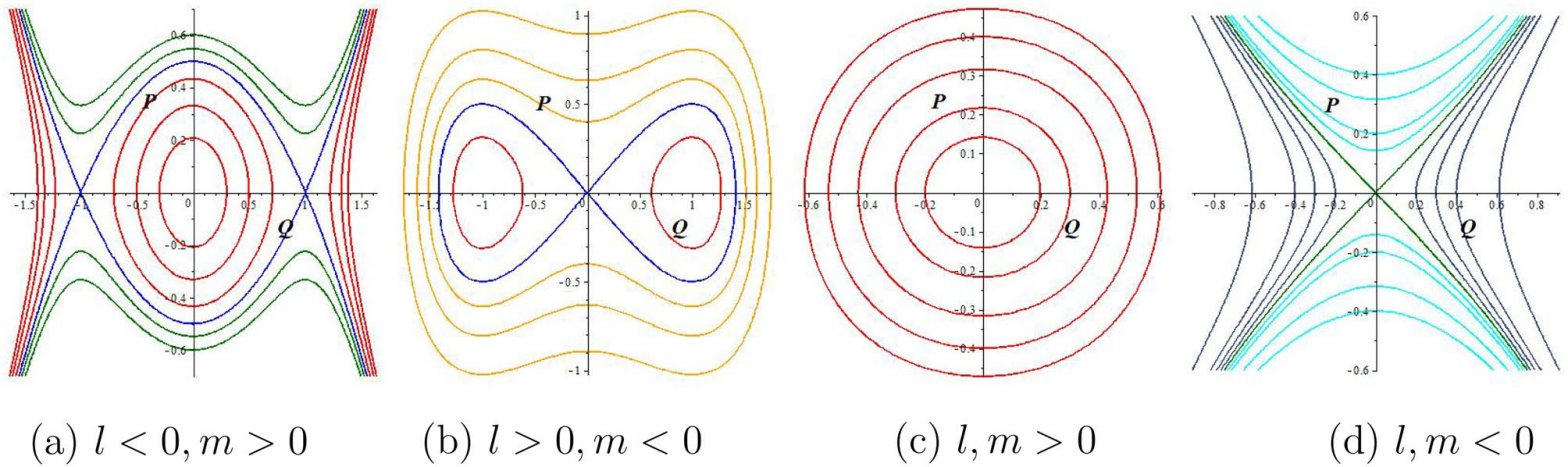
Visualization of phase diagrams of the dynamical system Eq. (12).

## Bifurcation analysis

This segment introduces the bifurcation and phase portraits of the next planner dynamical system. Nonlinear partial differential models can be analyzed qualitatively using this dynamic system method. The orbits of this system can manifest as points, simple closed curves, or other isomorphic curves. These varied orbits correspond to the solutions of Eq. (7) with distinct physical characteristics. Consider $\frac{dQ}{d\theta }=P$, then the planner dynamical form of Eq. (7) with Hamiltonian function can be written as

12 $$\begin{aligned} & \frac{dQ}{d\theta }=P, \frac{dP}{d\theta }=-lQ^3-mQ, \end{aligned}$$

13 $$\begin{aligned} & H(Q,P)=\frac{1}{2}P^2+\frac{l}{4}Q^4+\frac{m}{2}Q^2=h, \end{aligned}$$

where $l=\frac{a_2}{a_1a_3a_4}, m=\frac{a_1-a_4}{a_1a_3a_4}$, and *h* is the Hamiltonian constant.

Let $Q(\theta )$ be a solution to Equation Eq. (12) with the conditions $\lim _{\theta \rightarrow -\infty }Q(\theta )=u_1$ and $\lim _{\theta \rightarrow +\infty } Q(\theta )=u_2$, where $u_{1}$ and $u_{2}$ are constants. In the case where $u_1=u_2$, $Q(\theta )$ signifies a homoclinic orbit, leading to $Q(\theta )$ emerging a solitary wave solution of Eq. (11). Conversely, if $u_{1}\ne u_{2}$, then $Q(\theta )$ corresponds to a heteroclinic orbit. Specifically, for $u_1>u_2$, $Q(\theta )$ takes the form of a kink wave solution, while for $u_1<u_2$, it becomes an anti-kink wave solution. Another scenario arises when Eq. (12) exhibits a closed phase portrait, resulting in Eq. (11) having a periodic solution. It is noted that a phase portrait represents a collection of orbits in a phase plane.

For finding the equilibrium points of system Eq. (12), we solve the system of equations $P=0, -lQ^3-mQ=0$. Then only one equilibrium point (0, 0) is found for $lm>O$. On the other hand, three equilibrium points $(0,0), \left( \sqrt{-\frac{m}{l}},0\right)$, and $\left( -\sqrt{-\frac{m}{l}},0\right)$ are obtained for $lm<O$.

System Eq. (12) has a Jacobian matrix with the following determinant form

14 $$\begin{aligned} D(Q,P) =\begin{vmatrix} 0&0 \\ -3lQ^2-m&0 \end{vmatrix}=3lQ^2+m. \end{aligned}$$

Therefore, Eq. (12) has the characteristic value $\sqrt{-3lQ^2-m}$ at position (*Q*, 0). Consequently, the equilibrium point (*Q*, 0) accounts for a center point when *D*(*Q*, *P*) is positive, a saddle point when *D*(*Q*, *P*) is negative, and a cuspidal point when $D(Q, P)=0$. Various parameters can lead to the following possible outcomes:

*Case 1:* $$l<0, m>0$$

By selecting a parameter set such that $a_1=2,a_3=a_4=1, a_2=-1$, three equilibrium points (0, 0), (1, 0) and $(-1,0)$, are identified, as presented in Fig. 1a. Clearly, (0, 0) represents a center point, whilst (1, 0) and $(-1,0)$ correspond to saddle points. Fig. 1a demonstrates the existence of anti-kink and kink wave outcomes through the connection of two heteroclinic orbits $(-1,0)$ and (1, 0).

*Case 2:* $$l>0, m<0$$

By selecting a parameter set such that $a_1=a_2=a_3=1,a_4=2$, there exist three equilibrium points (0, 0), (1, 0), and (− 1,0)﻿ are identified, as presented in Fig. 1b. Evidently, (0, 0) represents a saddle point, whilst (1, 0) and $(-1,0)$ correspond to center points. The trajectories comprise closed curves, encompassing diverse solutions such as hyperperiodic (yellow curve), periodic (red curve), and homoclinic orbits (blue curve).

*Case 3:* $$l>0, m>0$$

By selecting a parameter set such that $a_2=-1,a_1=a_3=1,a_4=2$, one equilibrium point (0, 0), is identified, as presented in Fig. 1c. In this scenario, (0, 0) represents a center point. Figure Fig. 1c contains only one family of periodic orbits that can be obtained from the system Eq. (12).

*Case 4:* $$l<0, m<0$$

By selecting a parameter set such that $a_1=2,a_3=a_4=1, a_2=-1$, there is only one equilibrium point (0, 0), is identified, as presented in Fig. 1d. In this scenario, (0, 0) represents a saddle point. We see that there is no closed trajectory for the system Eq. (12).

**Figure 2:**
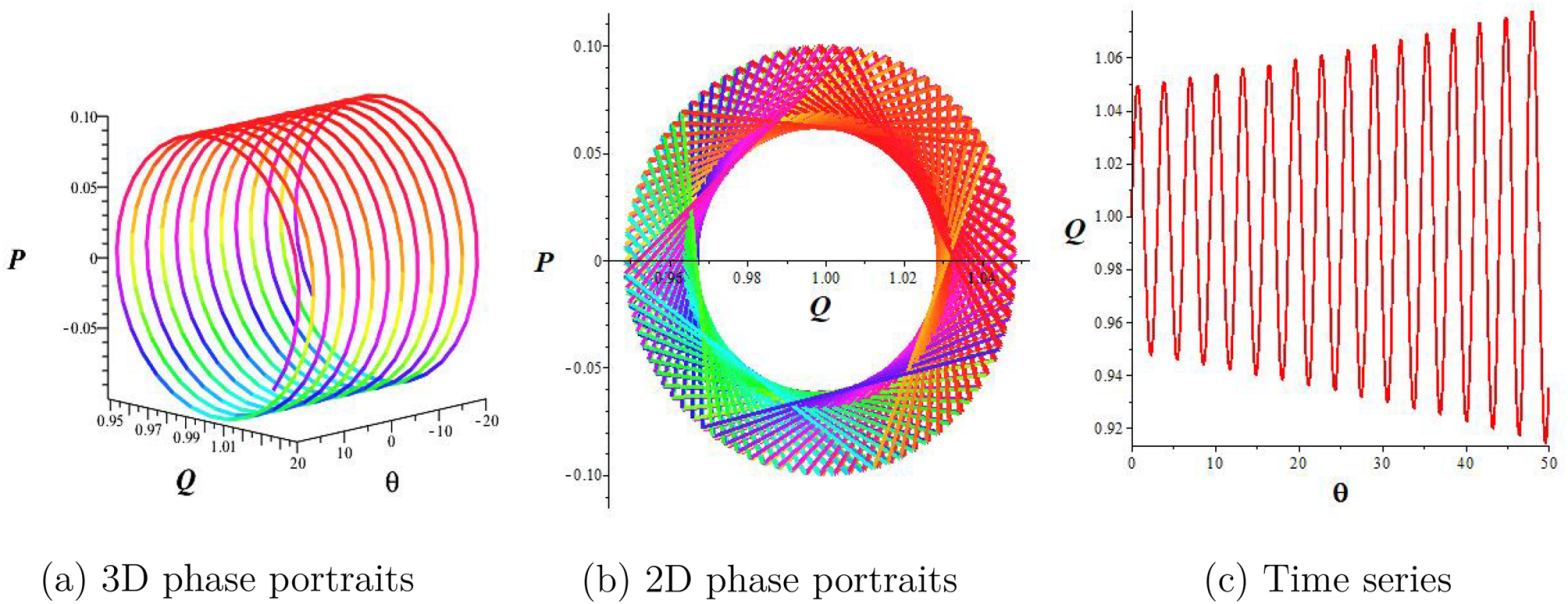
Periodic behavior of the structure Eq. (15) for $a_1=a_2=1,a_3=\frac{1}{4},$ $a_4=2,$ and $\sigma =0$.

## Chaotic behaviors

**Figure 3:**
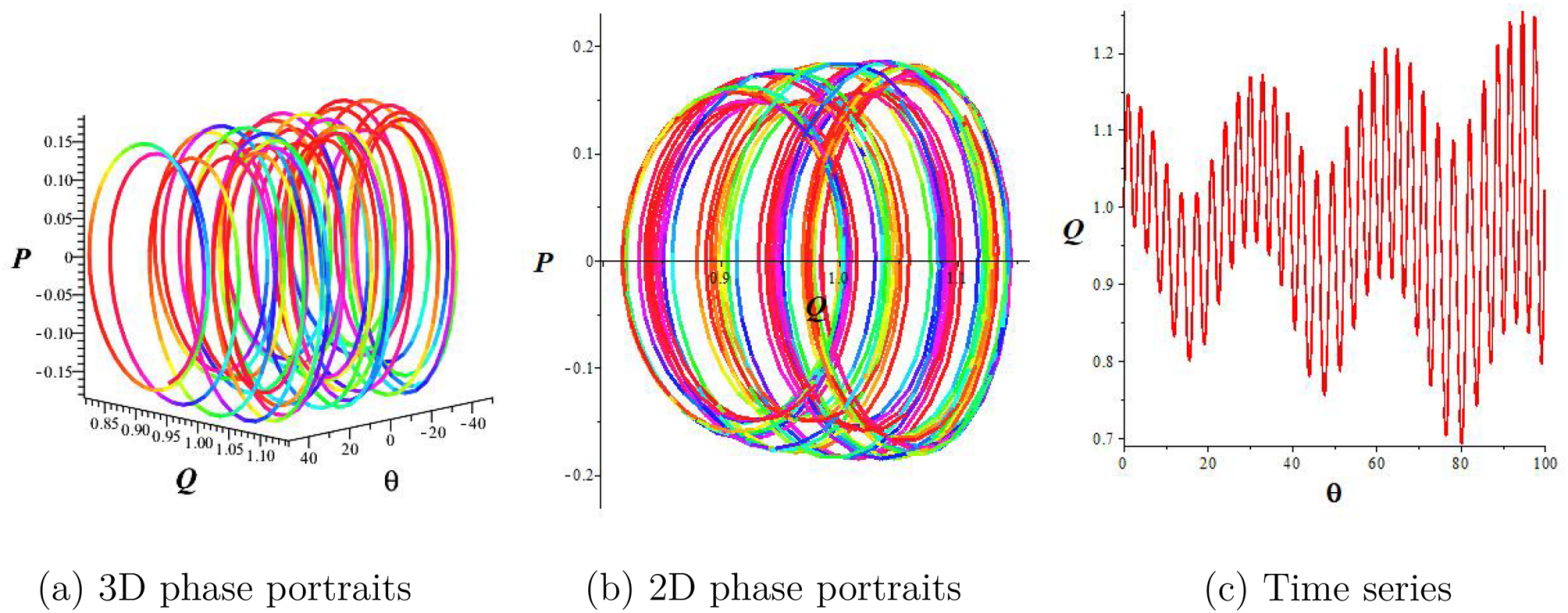
Quasi-periodic behavior of the structure Eq. (15) for $a_1=a_2=1,a_3=\frac{1}{4},$ $a_4=2,$ $\sigma =0.3,$ and $\omega =0.2$.

**Figure 4:**
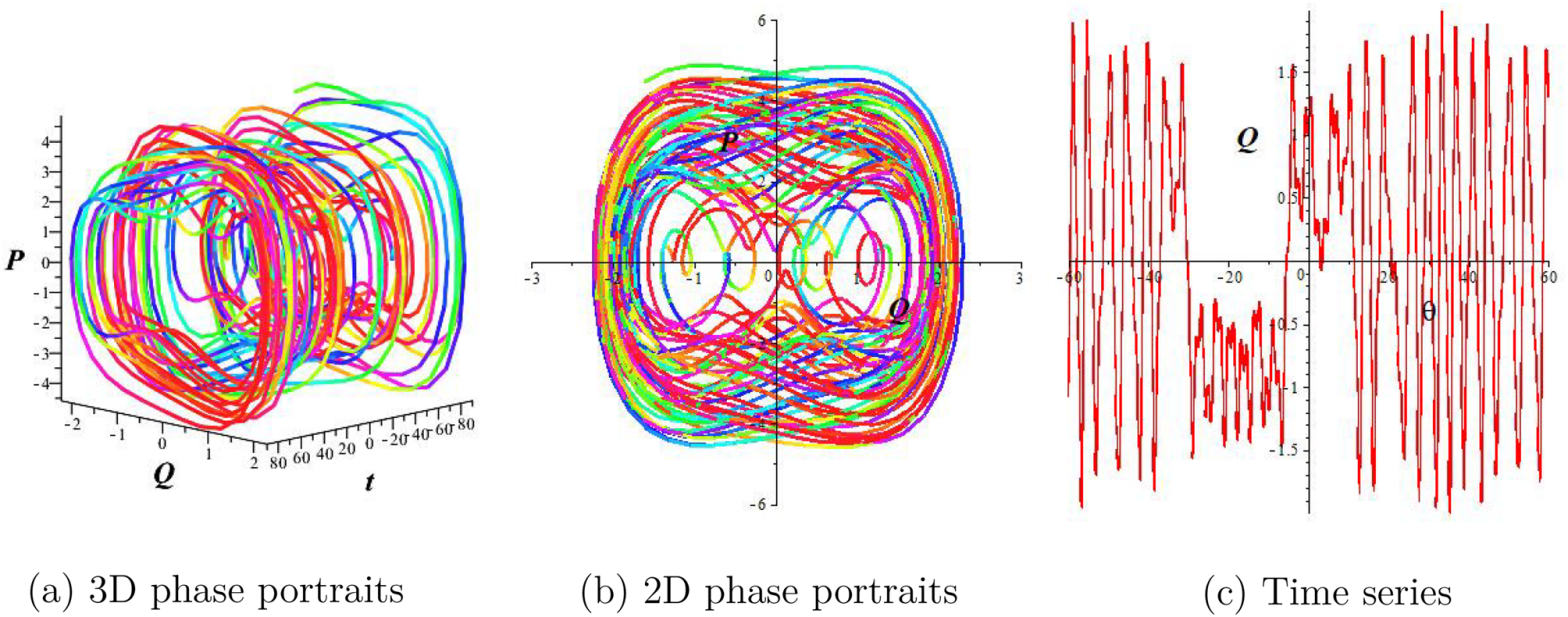
Chaotic behavior of the structure Eq. (15) for $a_1=a_2=1,a_3=\frac{1}{4},a_4=2,$ $\sigma =2.9,$ and $\omega =3.9$.

This section examines the chaotic features of the resulting dynamical system by considering perturbed terms. This analysis is conducted through the examination of 2D and 3D phase portraits. To initiate this investigation, let us consider the dynamical system:

15 $$\begin{aligned} & \frac{dQ}{d\theta }=P, \frac{dP}{d\theta }=-lQ^3-mQ+\sigma \cos (\omega \theta), \end{aligned}$$

where $\sigma \cos (\omega \theta)$ represents the perturbed term, $\sigma$ corresponds to the amplitude, and $\omega$ is the frequency of the system. In this section, we explore how the perturbation’s intensity and frequency impact the system Eq. (15). Keeping the main parameters fixed ($a_1=a_2=1,a_3=\frac{1}{4},a_4=2$), we obtain quasiperiodic and chaotic behaviors for diverse strengths and frequencies in Figs. 2, 3, and 4. Figure 2 signifies the state of Eq. (15) when $\sigma =0$. We display the trajectory’s status depending on the perturbation strength and frequency. Figure 2 displays the periodic nature of the system Eq. (15) in time series, 2D-, and 3D phase projections. The results of Fig. 3 show that the dynamic system changes from a period to a quasi-period with a small variation in strength and frequency ($\sigma$ increases to 0.3 and $\omega =0.2$). In Fig. 4, with increased frequency and strength ($\sigma$ increases to 2.9 and $\omega =3.9$), the system undergoes violent disturbances, transitioning into a chaotic state.

**Figure 5:**
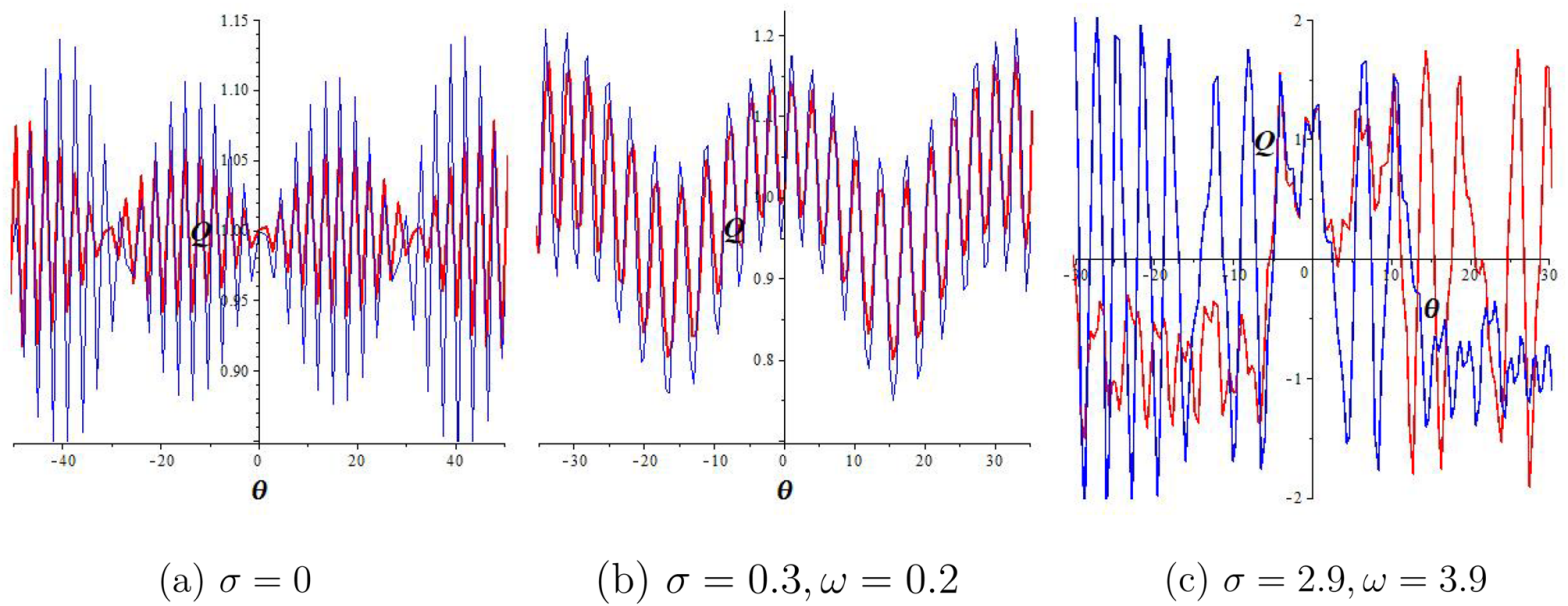
Sensitivity of equation Eq. (15) for initial values (1, 0.1) (red curve) and (1, 0.2) (blue curve) for $a_1=a_2=1,a_3=\frac{1}{4},$ and $a_4=2$.

## Sensitivity analysis

This portion investigates the impact of initial values on the perturbed system Eq. (15) across a range of strengths and frequencies, maintaining constant parameter values ($a_1=a_2=1,a_3=\frac{1}{4},a_4=2$). The results, depicted in Fig. 5, showcase a red curve representing a time series plot with initial values $(Q(0), P(0))=(1,0.1)$ and a blue curve with $(Q(0), P(0))=(1,0.2)$. In Fig. 5a, it is evident that the periodic nature of the outcome is determined by the initial value of the perturbed system  ($\sigma =0$)﻿﻿. Figure 5b illustrates that with a small perturbation strength ($\sigma =0.3$), the two-time series diagrams exhibit only small changes, indicating low sensitivity to the initial condition. Conversely, when the perturbation strength increases ($\sigma =2.9$), Fig. 5c reveals major changes between time series diagrams, signifying heightened sensitivity to changes in the initial value.

## Bright and dark solitons of the WBBM model

This section examines dark and bright solitons obtained from the mentioned model employing the planner dynamical system technique.

*Case 1:* $$l<0,$$ and $$m>0$$

For $h\in (0, -\frac{m^2}{4l})$, one can obtain a class of periodic orbits of the dynamical structure Eq. (12). In this scenario, the Hamiltonian system will be written in the next formation

16 $$\begin{aligned} & P=\pm \sqrt{-\frac{l}{2}}\sqrt{Q^4+\frac{2m}{l}Q^2-\frac{4h}{l}}=\pm \sqrt{-\frac{l}{2}}\sqrt{(\phi _1^2-Q^2)(\phi _2^2-Q^2)}, \end{aligned}$$

where $$\phi _1=\sqrt{-\frac{m}{l}+\frac{\sqrt{m^2+4lh}}{l}}$$ and $$\phi _2=\sqrt{-\frac{m}{l}-\frac{\sqrt{m^2+4lh}}{l}}$$.

By employing Eq. (16) into the first equation of the Hamiltonian structure Eq. (12), and integrating we arrive

17 $$\begin{aligned} \int ^Q_0 \frac{dR}{\sqrt{(\phi _1^2-R^2)(\phi _2^2-R^2)}}=\pm \sqrt{-\frac{l}{2}}(\theta -\theta _0), \end{aligned}$$

with integral constant $\theta _0$.

Therefore, we obtain the next two periodic wave outcomes as

$$\begin{aligned} q_1(x,t)=\pm \phi _1 sn(\phi _2 \sqrt{-\frac{l}{2}}(\frac{a_1}{\alpha }x^\alpha +\frac{a_2}{\alpha }y^\alpha +\frac{a_3}{\alpha }z^\alpha -\frac{a_4}{\alpha }t^\alpha -\theta _0),\frac{\phi _1}{\phi _2}), \end{aligned}$$

For $h=-\frac{m^2}{4l}$, we have $\phi _1^2=\phi _2^2=-\frac{m}{l}$, and the next kink wave (for positive sign) and antikink wave (for negative sign) solutions are obtained

$$\begin{aligned} q_2(x,t)=\pm \sqrt{-\frac{m}{l}} \tanh \left( \sqrt{\frac{m}{2}}\left( \frac{a_1}{\alpha }x^\alpha +\frac{a_2}{\alpha }y^\alpha +\frac{a_3}{\alpha }z^\alpha -\frac{a_4}{\alpha }t^\alpha -\theta _0\right) \right) , \end{aligned}$$

*Case 2:* $$l>0,$$ and $$m<0$$

For $h\in (-\frac{m^2}{4l}, 0)$, one can acquire two classes of periodic orbits of the dynamical structure Eq. (12). In this case, the Hamiltonian system will be written in the subsequent formation

18 $$\begin{aligned} & P=\pm \sqrt{\frac{l}{2}}\sqrt{-Q^4-\frac{2m}{l}Q^2+\frac{4h}{l}}=\pm \sqrt{\frac{l}{2}}\sqrt{(Q^2-\phi _1^2)(\phi _2^2-Q^2)}, \end{aligned}$$

where $$\phi _1=\sqrt{-\frac{m}{l}+\frac{\sqrt{m^2+4lh}}{l}}$$ and $$\phi _2=\sqrt{-\frac{m}{l}-\frac{\sqrt{m^2+4lh}}{l}}$$.

By employing Eq. (18) into the first equation of the Hamiltonian structure Eq. (12), and integrating we arrive

19 $$\begin{aligned} \int ^{\phi _2}_Q \frac{dR}{\sqrt{(R^2-\phi _1^2)(\phi _2^2-R^2)}}=\mp \sqrt{\frac{l}{2}}(\theta -\theta _0), \end{aligned}$$

and

20 $$\begin{aligned} \int ^Q_{-\phi _2} \frac{dR}{\sqrt{(R^2-\phi _1^2)(\phi _2^2-R^2)}}=\pm \sqrt{\frac{l}{2}}(\theta -\theta _0), \end{aligned}$$

then we acquired the next two periodic wave outcomes as

$$\begin{aligned} q_3(x,t)=\pm \phi _1 dn\left( \phi _1 \sqrt{\frac{l}{2}}\left( \frac{a_1}{\alpha }x^\alpha +\frac{a_2}{\alpha }y^\alpha +\frac{a_3}{\alpha }z^\alpha -\frac{a_4}{\alpha }t^\alpha -\theta _0\right) ,\frac{\sqrt{\phi _1^2-\phi _2^2}}{\phi _1}\right) , \end{aligned}$$

For $h=0$, we have $\phi _2=0$ and $\phi _1=\sqrt{-\frac{2m}{l}}$, and the next two bright (for positive sign) and dark (for negative sign) bell solitary wave outcomes are obtained

$$\begin{aligned} q_4(x,t)=\pm \sqrt{-\frac{2m}{l}} {\text {sech}}\left( \sqrt{-m} \left( \frac{a_1}{\alpha }x^\alpha +\frac{a_2}{\alpha }y^\alpha +\frac{a_3}{\alpha }z^\alpha -\frac{a_4}{\alpha }t^\alpha -\theta _0\right) \right) , \end{aligned}$$

For $h\in (0, +\infty )$, the Hamiltonian system will be written in the following way

21 $$\begin{aligned} & P=\pm \sqrt{\frac{l}{2}}\sqrt{-Q^4-\frac{2m}{l}Q^2+\frac{4h}{l}}=\pm \sqrt{\frac{l}{2}}\sqrt{(\phi _1^2-Q^2)(\phi _3^2+Q^2)}, \end{aligned}$$

where $$\phi _1=\sqrt{-\frac{m}{l}+\frac{\sqrt{m^2+4lh}}{l}}$$ and $$\phi _3=\sqrt{\frac{m}{l}+\frac{\sqrt{m^2+4lh}}{l}}$$.

By employing Eq. (21) into the first equation of the Hamiltonian structure Eq. (12), and integrating we arrive

22 $$\begin{aligned} \int ^Q_0 \frac{dR}{\sqrt{(\phi _1^2-R^2)(\phi _3^2+R^2)}}=\pm \sqrt{\frac{l}{2}}\left(\theta -\theta _0\right) , \end{aligned}$$

then we acquired the next periodic wave outcomes as

$$\begin{aligned} q_5(x,t)=\pm \phi _1 cn\left( \sqrt{\frac{l(\phi _1^2+\phi _3^2)}{2}}\left( \frac{a_1}{\alpha }x^\alpha +\frac{a_2}{\alpha }y^\alpha +\frac{a_3}{\alpha }z^\alpha -\frac{a_4}{\alpha }t^\alpha -\theta _0\right) ,\frac{\phi _1}{\sqrt{\phi _1^2+\phi _3^2}}\right) . \end{aligned}$$

**Figure 6:**
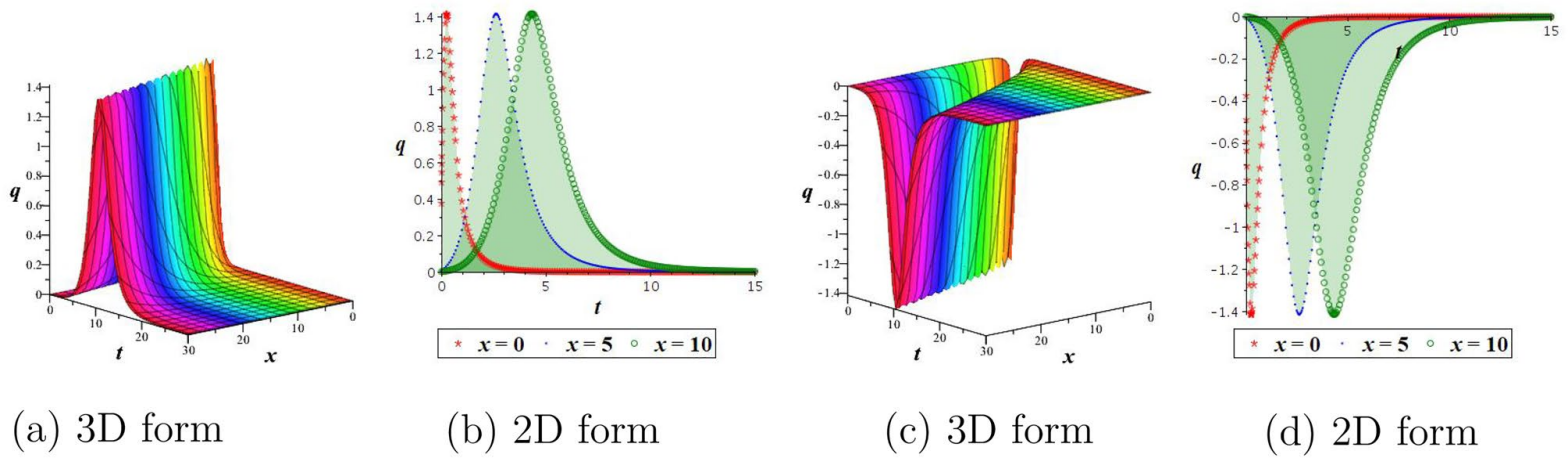
Outlook of outcome $q_4$: (**a**,**b**) bright soliton, (**c**,**d**) dark soliton.

**Figure 7:**
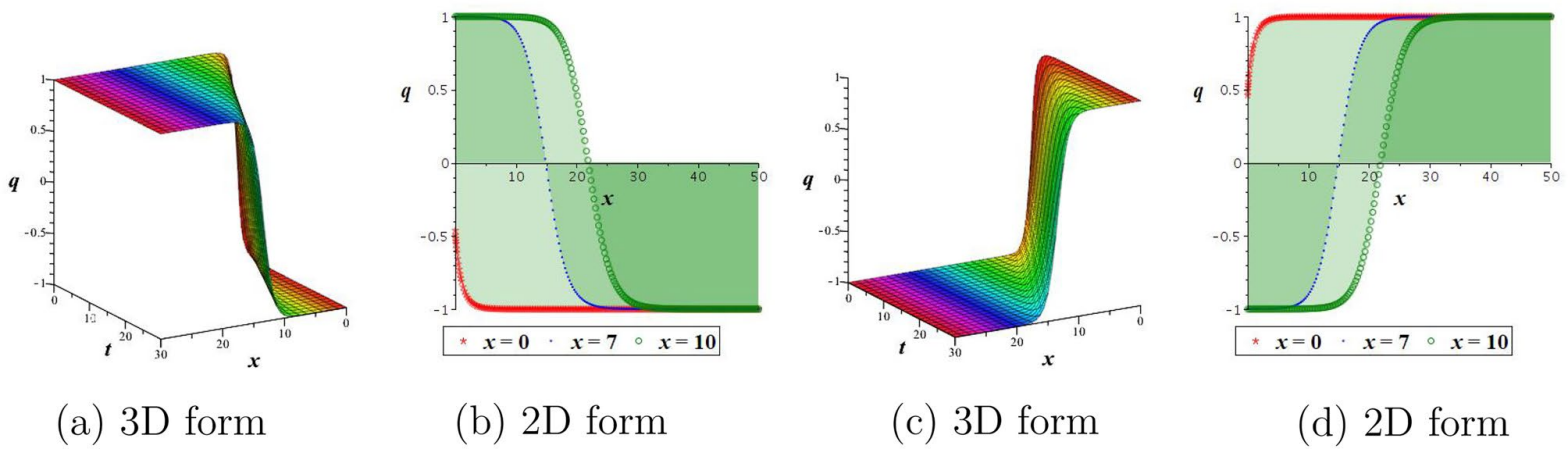
Outlook of outcome $q_2$: (**a**,**b**) kink wave, (**c**,**d**) anti-kink wave.

**Figure 8:**
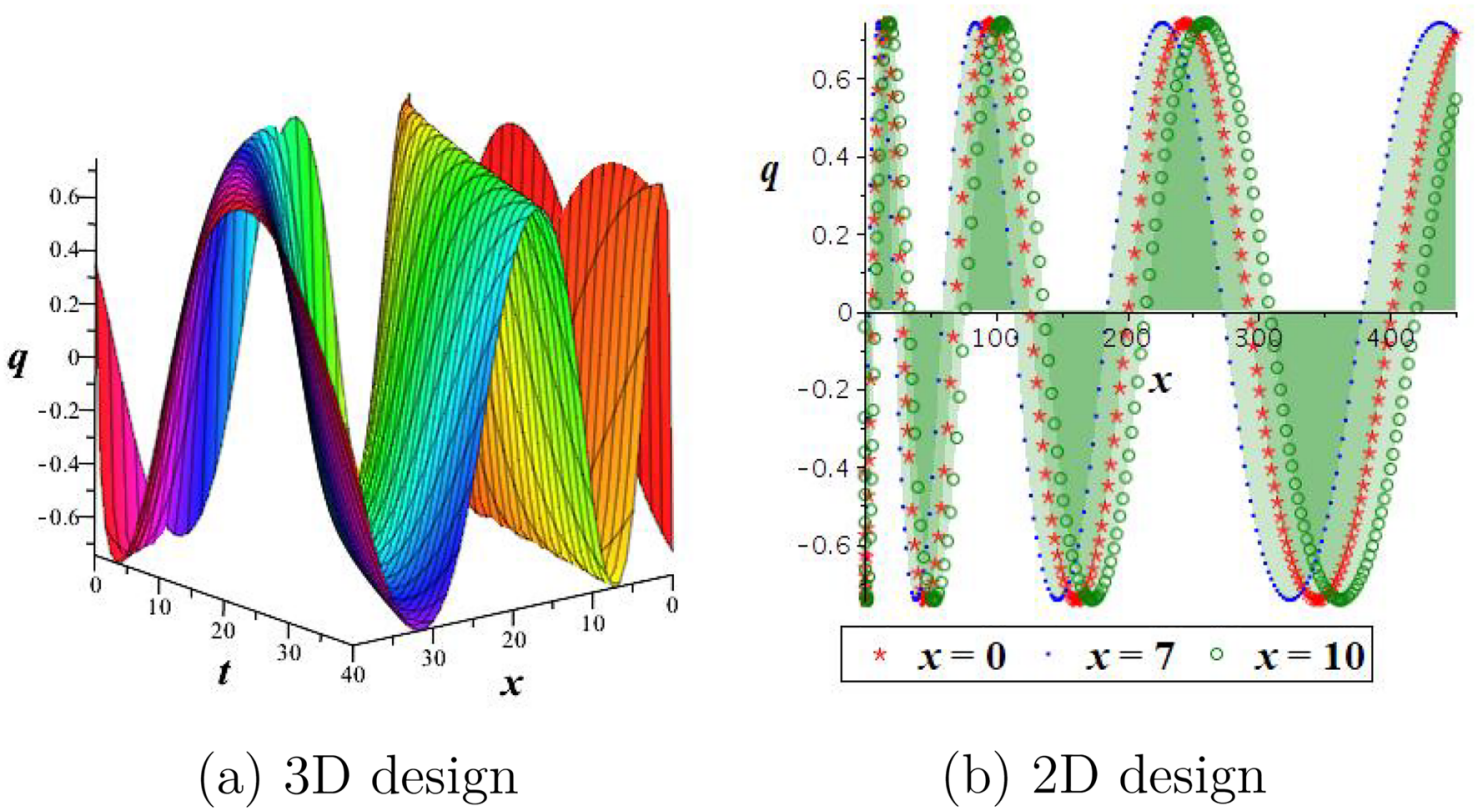
Outlook of periodic wave outcome $q_1$.

## Discussion of results

Using appropriate parameter values, we describe numerical simulations of the obtained outcomes and give their physical interpretation. For solution $q_4$, the positive sign is displayed with a bright soliton, while the negative sign is displayed with a dark soliton. It is shown in Fig. 6 how the physical nature of the precise outcome $q_4$ will change when $\theta _0=1, \alpha =0.5, a_1=a_2=1,a_3=\frac{1}{2},$ and $a_4=2$. It is observed that for the positive sign, Fig. 6a,b is signified by a bright soliton, while for the negative sign, Fig. 6c,d is signified by a dark soliton. To examine the physical properties of the precise outcome, $q_2$, a numerical simulation is presented in Fig. 7 for the parameters $\theta _0=1, \alpha =0.8, a_1=2,a_2=-1,a_3=a_4=1,$ and $h=\frac{1}{8}$. One can observe from the figure that for the positive sign, Fig. 7a,b is signified by a kink wave, while for the negative sign, Fig. 7c,d is signified by an anti-kink wave. Solutions $q_1, q_3$, and $q_5$ exhibit a periodic wave. Finally, to examine the physical properties of the precise outcome, $q_1$, a numerical simulation is presented in Fig. 8 for the parameters $\theta _0=1, \alpha =0.5, a_1=2,a_2=-1,a_3=a_4=1,$ and $h=\frac{1}{10}$.

**Figure 9:**
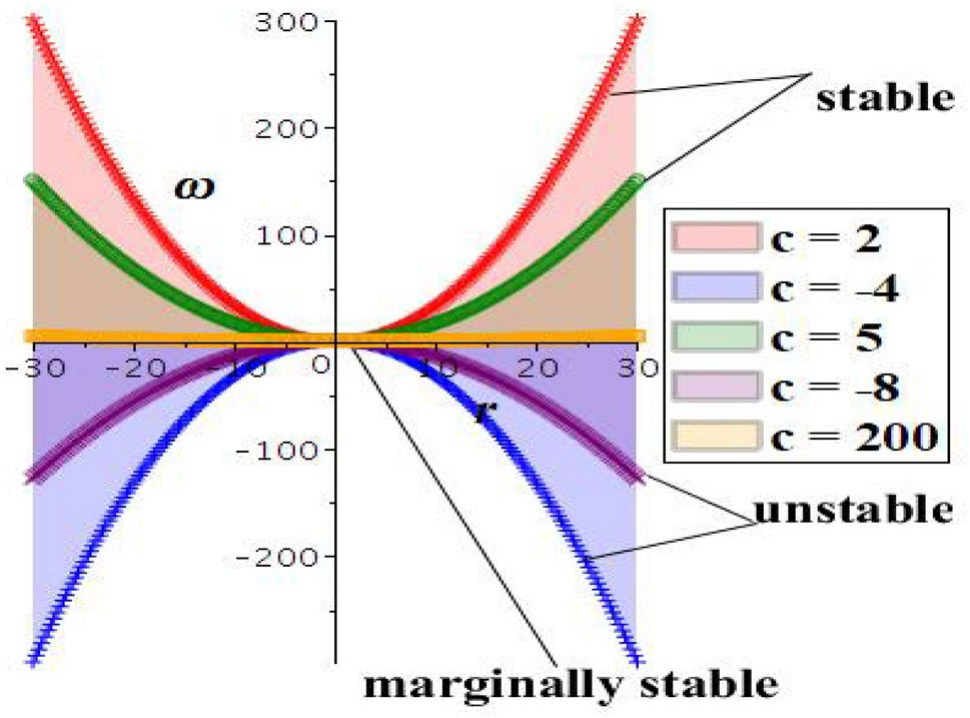
Outlook of the stability graphic for the perturbation Eq. (28), with $d=e=1$.

## Stability analysis

To analyze the stability of the WBBM equation, this research will conduct linear stability analysis, as described in^38^. Assume WBBM’s integer order, as stated in Eq. (4). Now, the perturbation solution takes the following structure:

23 $$\begin{aligned} q(x,y,z,t)=r+\lambda s(x,y,z,t), \end{aligned}$$

with incident power *r*. Inserting equation Eq. (23) into Eq. (4), one reaches

24 $$\begin{aligned} \lambda (s_ys^2 \lambda ^2+2s_y s \lambda r+s_y r^2+s_t+s_x-s_{zxt})=0, \end{aligned}$$

By linearizing the immediate equation in the form $\lambda$ reads,

25 $$\begin{aligned} s_yr^2+s_t+s_x-s_{zxt}=0, \end{aligned}$$

Now, we suppose that the next solution to the above equation

26 $$\begin{aligned} s(x,y,z,t)=e^{i(cx+dy+ez-\omega t)}, \end{aligned}$$

with normalized wave number *c*, *d*, *e* and frequency $\omega$. Plugging Eq. (26) into Eq. (25) reads

27 $$\begin{aligned} \omega ce-dr^2+\omega -c=0, \end{aligned}$$

By solving the above equation one can reach the value of $\omega$

28 $$\begin{aligned} \omega (c,d,e)=\frac{c+dr^2}{ce+1}, ce\ne -1 \end{aligned}$$

The examination delves into the analysis of propagation relationships Eq. (28) as described in Fig. 9. Figure shows that the positive sign of $\omega (c, d, e)$ indicates whether the solution will amplify or diminish over time. The incident power r and wave numbers *c*, *d*,  and *e* are stable states for small perturbations (red and green curves). The system remains marginally stable when $\omega (c, d, e) = 0$, as disturbances do not grow or decay over time (yellow curve). Additionally, if $\omega (c, d, e)$ is negative, the system moves further away from equilibrium over time, leading to instability in steady-state solutions and exponential distortion growth (blue and purple curves).

## Novelty of the outcomes

This paragraph compares our results with recent studies, illustrating our findings’ novelty. A literature review cited in^56–59^ is included to determine the originality of our results. Mamun and his collaborators presented solitary and periodic wave solutions of the suggested model taking advantage of the $(G'/G^2)$-expansion process^56^. Akram and his coauthors obtained some traveling wave solutions to this model employing the EMAEM method^57^. Inc and his colleagues solved this model through the Sarder-subequation scheme^58^. Kaabar and others investigated the suggested model utilizing the generalized Kudryashov process and $\exp (-\phi (\zeta ))$ technique^59^. Our results $q_1, q_2, q_3, q_4$, and $q_5$ exhibit novelty, when compared to their corresponding solutions. Notably, stability, bifurcation, chaos, and sensitivity analysis of the governing model have not been reported in the literature. Consequently, our comparison highlights the novelty of additional solutions, representing the first instances of their construction for the investigated model.

## Conclusion

We have effectively investigated bifurcation analysis and new waveforms to the first fractional 3D WBBM equation that appeared in shallow water waves. Moreover, the linear stability process is performed to assess the model’s stability. By implementing the Galilean transformation, we have successfully obtained the dynamical system of the mentioned model, facilitating a comprehensive bifurcation analysis. Additionally, we explored various solitary wave solutions, including dark solitons, bright solitons, kink waves, periodic waves, and anti-kink waves. Through simulations, we visually presented these solutions, emphasizing their distinct characteristics and existence. The results highlight the effectiveness, brevity, and efficiency of the employed integration methods. They also suggest their applicability to delving into more intricate nonlinear models emerging in modern science and engineering scenarios.

## Data Availability

All data generated or analysed during this study are included in this article.
